# The Approaching End of Boards of Guardians

**Published:** 1919-03-15

**Authors:** 


### THE APPROACHING END OF BOARDS OF GUARDIANS.

Poor-Law Guardians in conference at the Guildhall recently discussed the Ministry of Health and its effect upon themselves. As was to be expected, most of the speakers strongly denounced any change in the present system: a few approved and welcomed the proposed reforms. Our Poor-law system to-day deals with two matters it is important to keep apart. The one is the medical treatment of the sick poor, the other is the relief of those who are in need and who are healthy. Upon the first the medical profession can give an expert opinion and expert advice; the latter concerns its members to the same extent only as it concerns all citizens of the State.

No one will deny that recently, and especially since the Report of the Royal Commission on the Poor-Law, most Boards of Guardians have improved their methods of dealing with the sick poor. The fact remains that, owing to the indifference of Guardians, to their fear of an immediate expenditure to gain a remote benefit, and to defects inherent in the Poor-Law system, the equipment and staffing of the Poor-Law infirmaries lag far behind those of the voluntary hospitals. In proof of this statement it is sufficient to point out that not one of the large Poor-Law infirmaries with 500 to 1,000 beds has the specialist staff found to be essential by voluntary hospitals with 200 or 300 beds, that there are large infirmaries without an $x$-ray apparatus, without a theatre for operations, without a pathological and bacteriological department, and without the means of dealing adequately with the large number of paralysed and crippled patients housed within them. Boards of Guardians are running their infirmaries to-day on lines that would have been adequate forty years ago, but which the advance in specialised methods of treatment and diagnosis has put completely out of date. This increase in the complexity of medical work renders it impossible to deal properly and economically with all the sick poor of one parish in one infirmary. We want for our sick poor clearing hospitals, in which patients will be sorted and special cases sent to hospitals specially equipped and staffed for their treatment. Above all, we want the curative work brought into close touch with the system of preventive medical work and with an organised system of medical research. To obtain these needs the treatment of the sick poor must be dealt with administratively over large areas, and the work must be correlated with the work of the county medical officer of health and with the other public medical work in the county.

